# HIV is a virus, not a crime: ten reasons against criminal statutes and criminal prosecutions

**DOI:** 10.1186/1758-2652-11-7

**Published:** 2008-12-01

**Authors:** Edwin Cameron, Scott Burris, Michaela Clayton

**Affiliations:** 1 Supreme Court of Appeal of South Africa, Bloemfontein, South Africa; 2 Temple University Beasley School of Law, Philadelphia, USA; 3 AIDS and Rights Alliance for Southern Africa, Windhoek, Namibia

## Abstract

The widespread phenomenon of enacting HIV-specific laws to criminally punish transmission of, exposure to, or non-disclosure of HIV, is counter-active to good public health conceptions and repugnant to elementary human rights principles. The authors provide ten reasons why criminal laws and criminal prosecutions are bad strategy in the epidemic.

## Debate

Criminalisation of HIV transmission has become a pressing issue in the management of the epidemic. Let us illustrate by referring to three vivid instances, from Texas, Zimbabwe and Sierra Leone.

In Texas in May 2008, a homeless man was sent to jail. He was convicted of committing a serious offence while being arrested for drunk and disorderly conduct – namely, harassing a public servant with a deadly weapon. Because of his past encounters with the law, the system ratcheted up the gravity of what he did, and he ended up being sentenced to 35 years in jail – of which he must serve at least half before he can apply for parole [1].

The man had HIV. The 'deadly weapon' he used against the public servant was his saliva. He was jailed because he spat at the officers who were arresting him. After sentencing, Officer Waller is reported to have said he was elated with the jury's decision: 'I know it sounds cliché [d], but this is why you lock someone up...Without him out there, our streets are a safer place.' [2].

This was an overstated claim. First, according to the most assured scientific knowledge we have, after nearly three decades of studying the virus, saliva 'has never been shown to result in transmission of HIV' [3]. So the 'deadly weapon' the man was accused of wielding was no more than a toy pistol – and it wasn't even loaded. Ratcheting up the criminal law because the man had HIV was thus inappropriate, unscientific and plain wrong.

Second, the length of the sentence is ferocious. Whatever his past conduct, it stuns the mind that someone who has not actually harmed anyone or damaged any property (or otherwise spoiled the world) could be locked away for 35 years. The inference that his HIV status played a significant, probably pivotal, part in sending him away for so long is unavoidable. In short: the man was punished not for what he did, but for the virus he carried.

In Zimbabwe, a 26-year-old woman from a township near Bulawayo was arrested last year for having unprotected sex with her lover. Like the homeless Texan, she was living with HIV. The crime of which she was convicted was 'deliberately infecting another person'. Her lover however tested HIV negative. The woman was receiving antiretroviral therapy, so that is not entirely surprising [4]. Before sentencing her, the court tried to get a further HIV test from the lover – even though he reportedly didn't want to proceed with the charges at all [5]. She was eventually sentenced to a suspended term of five years' imprisonment [6]. The threat of imprisonment, and the shame and ordeal of her conviction, will continue to hang over her.

The statute under which she was convicted, s79 of the Zimbabwe Criminal Law (Codification and Reform) Act 23 of 2004, is an extraordinary piece of legislation. It doesn't make it a crime merely for a person who knows that she has HIV to infect another. It makes it a crime for anyone who realises 'that there is a real risk or possibility' that she might have HIV, to do 'anything' that she 'realises involves a real risk or possibility of infecting another person with HIV'.

In other words, though the crime is called 'deliberate transmission of HIV', this is a misnomer. You can commit this crime even if you do not transmit HIV. In fact, you can commit the crime even if you do not have HIV. You merely have to realise 'that there is a real risk or possibility' that you have HIV – and then do something – 'anything' – that involves 'a real risk or possibility of infecting another person'.

Stranger upon strange, this statute offers a defence when a person really does has HIV. In such a case, if the other person knew this, and consented, then the accused is exempt. But, the way the statute is drafted, this defence can not apply where the accused does not in fact have HIV, or does not know that she has HIV – by definition, in that case she cannot engage the informed consent defence by telling her partner she has HIV! In short, this law creates a crime not of effect and consequence, but of fear and possibility.

What is more, the wording of Zimbabwe law stretches wide enough to cover a pregnant woman who knows she has, or fears she may, have HIV. For if she does 'anything' that involves a possibility of infecting another person – like, giving birth, or breast-feeding her newborn baby – the law could make her guilty of 'deliberate transmission – even if her baby is not infected. In all cases, the law prescribes punishment of up to twenty years in prison.

In Sierra Leone, lawmakers have gone even further. They have avoided subtle lawyers' arguments about whether their law applies to pregnant women. So they have enacted a statute that removes all doubt. Their law also creates an offence of 'HIV transmission', though it too criminalises exposure to HIV, even without transmission.

The Sierra Leone law requires a person with HIV who is aware of the fact to 'take all reasonable measures and precautions to prevent the transmission of HIV to others' – and it expressly covers a pregnant woman [7]. It requires her to take reasonable measures to prevent transmitting HIV to her foetus. No one doubts a mother's will and duty to take reasonable steps to protect her baby, but the law will make it more difficult for her to do so.

In addition, a person with HIV who is aware of this fact must not knowingly or recklessly place another at risk of becoming infected with HIV, unless that person knew of the fact and voluntarily accepted the risk. This, too, applies to pregnant mothers.

The provision criminalises not merely actual transmission of HIV from mother to child, but makes a criminal of any pregnant woman who knows she has HIV but does not take reasonable measures to prevent transmission to her baby.

There are many other sad, vivid and frightening current examples:

• In Egypt, Human Rights Watch reports that men are being arrested merely for having HIV under article 9(c) of Law 10/1961, which criminalizes the 'habitual practice of debauchery [fujur]', a term used to penalize consensual homosexual conduct in Egyptian law [8].

• In Singapore, [9] a man with HIV has been sentenced to a year in prison for exposing a sexual partner to the virus even though the risk to the partner (whom he fellated) was minimal, if not non-existent.

• In Bermuda, a man with HIV who had unprotected sex with his girlfriend has been sentenced to ten years' imprisonment, even though he did not infect her [10].

• In June 2008, the highest court in Switzerland held a man liable for negligently transmitting HIV to a sexual partner when he knew that a past partner had HIV, even though he believed, because he experienced no seroconversion symptoms, that he himself did not have HIV [11].

These laws are stunningly wide in their application, and fearsome in their effects. They attack rational efforts to lessen the impact and spread of the epidemic with a sledge-hammer. They represent a rash phenomenon that is taking place world-wide:

• Law-makers are putting on the statute books new laws that create special crimes of HIV transmission or exposure. In Africa, the continent that carries the heaviest burden of the epidemic, at least a dozen countries [12] have already adopted laws similar to the Sierra Leone law (though not all of them expressly include pregnant women). Many countries have done so with the proud help of an American-funded organisation (See also additional file 1).

• Courts and prosecutors are targeting men and women with HIV for special prosecution.

These laws and prosecutions are creating a crisis in HIV management and prevention efforts, and they constitute one of the biggest threats to a rational and effective response to the epidemic. We should try to understand what lies behind criminalisation.

HIV is a fearsome virus, and its effects are potentially deadly. Public officials should be able to invoke any available and effective means to counter its spread. This includes criminal statutes and criminal prosecutions. Moreover, in the abstract and from a distance from social reality, there seems a certain justice that criminal penalties should be applied against those who negligently, recklessly or deliberately pass on the virus – even against those whose actions create only the risk of doing so.

African law-makers and policy-makers, in particular, have reason to look for strong remedies. Many African countries face a massive epidemic with agonising social and economic costs: all effective means, including the mechanisms of the criminal law and criminal prosecutions, must be utilised.

In addition, many law-makers are spurred especially by the plight of women. Many (including very young women) are infected by unwary or unscrupulous men. They need special protection, and some law-makers have concluded that a criminal statute may best give voice to their entitlement to protection.

It is true that the law can indeed play a constructive role in the response to HIV, especially in addressing the unequal and vulnerable position of many women. But the conclusion that HIV-specific criminal provisions and prosecutions should be part of the legal response is bad. And it should be countered, rationally, powerfully and systematically. We wish to offer the ten plainest reasons why criminal laws and criminal prosecutions make bad policy in the AIDS epidemic.

First, criminalisation is ineffective. These laws and prosecutions don't prevent the spread of HIV. In the majority of cases, the virus spreads when two people have consensual sex, neither of them knowing that one (who may be in the early, highly infectious stage during and soon after seroconversion) has HIV. That will continue to happen, no matter what criminal laws are enacted, and what criminal remedies are enforced. Criminalisation will not stand in the way of the vast majority of HIV transmissions.

Second, criminal laws and criminal prosecutions are a shoddy and misguided substitute for measures that really protect those at risk of contracting HIV. We know what we need in this epidemic. After more than a quarter-century, we know very well. We need effective prevention, protection against discrimination, reduced stigma, strong leadership and role models, greater access to testing, and, most importantly, treatment for those who, today, this morning, are unnecessarily dying of AIDS.

AIDS is now a medically manageable condition. It is a virus, not a crime, and we must reject interventions that suggest otherwise. We must focus on ending deaths, on ending stigma, on ending discrimination, and on ending suffering. And, we also must focus on ending irrational, unhelpful and resource-reducing measures like criminalisation.

For the uninfected, we need greater protection for women, more secure social and economic status, and we must enhance their capacity to negotiate safer sex and to protect themselves from predatory sexual partners. Criminal laws and prosecutions will not do that. What they do, instead, is to distract us from reaching that goal.

Third, far from protecting women, criminalisation victimises, oppresses and endangers them. In Africa most people who know their HIV status are female. This is because most testing occurs at ante-natal healthcare sites. The result, inevitably, is that most of those who will be prosecuted because they know – or ought to know – their HIV status will be women – like the Zimbabwe woman who now has a five-year prison sentence hanging over her.

As the International Community of Women Living with HIV/AIDS has pointed out in a powerful consultation process, many women cannot disclose their status to their partners because they fear violent assault or exclusion from the home. If a woman in this position continues a sexual relationship (whether consensually or not), she risks prosecution under the African model statutes for exposing her partner to HIV (even when she does not pass HIV on to him).

The material circumstances in which many women find themselves – especially in Africa – make it difficult, and all too often impossible, for them to negotiate safer sex, or to discuss HIV at all. These circumstances include social subordination, economic dependence and traditional systems of property and inheritance, which make them dependent on men.

These provisions will hit women hardest, and will expose them to assault, ostracism and further stigma. They will become more vulnerable to HIV, not less.

Fourth, criminalisation is often unfairly and selectively enforced. Prosecutions and laws single out already vulnerable groups – like sex workers, men who have sex with men and, in European countries, black males.

Women who are already marginalized, such as sex workers and drug users, are placed at risk of further targeting by government officials and agencies. This targeting is made more acute by the fact that, thus far, these laws have been relatively rarely applied. Such prosecutions as there have been have resulted from individual and sometimes idiosyncratic decisions by particular police officers and prosecutors. The fact is that, if we leave aside cases of deliberate transmission of HIV, the behaviour that is prosecuted – namely, sex between two consenting adults – is common. The prosecutions have therefore been necessarily arbitrary.

Fifth, criminalisation places blame on one person instead of responsibility on two. This is a hard but important thing to say. HIV has been around for nearly three decades. For nearly three decades the universal public information message has been that no one is exempt from it. So the risk of getting HIV (or any sexually transmitted infection) must now be seen as an inescapable facet of having sex. We cannot pretend that the risk is introduced into an otherwise safe encounter by the person who knows or should know he has HIV. The risk is part of the environment, and practical responsibility for safer sex practices rests on everyone who is able to exercise autonomy in deciding to have sex with another.

The person who passes on the virus may be 'more guilty' than the person who acquires it, but criminalisation unfairly and inappropriately places all the 'blame' on the person with HIV. It is true (as pointed out earlier) that the subordinate position of many women makes it impossible for them to negotiate safer sex. When a woman has no choice about sex, and gets infected, her partner unquestionably deserves blame. But the fact is that criminalisation does not help women in this position. It simply places them at greater risk of victimisation. Criminalisation singles one sexual partner out. All too often, despite her greater vulnerability, it will be the woman. Criminalisation compounds the evil, rather than combating it.

Sixth, these laws are difficult and degrading to apply. This is because they intrude on the intimacy and privacy of consensual sex. Nor are we talking about non-consensual sex. That is rape, and rape should always be prosecuted. But where sex is between two consenting adult partners, the apparatus of proof and the necessary methodology of prosecution degrade the parties and debase the law. The Zimbabwean woman again springs to our attention: her lover wanted the prosecution withdrawn, but the law vetoed his wishes. It also countermanded her interests. The result is a tragedy for all, and a blight on HIV prevention and treatment efforts.

What is more, the legal concepts of negligence and even recklessness are often incoherent in the realm of sexual behaviour, and incapable of truly just application. No one suggests that a person knowing he has HIV, who sets out intending to infect another, and achieves his aim, ought to escape prosecution (such as deliberately stabbing someone with an injecting needle containing blood with HIV) [13]. He has set out deliberately to harm another and he has achieved his purpose as surely as if he had wounded his victim with a firearm or a knife. In all these cases, the victims and their society seek justice because harm was caused with clear intention.

But in cases where there is no deliberate intention, the categories and distinctions of the criminal law become fuzzy and incapable of offering clear guidance – to those affected by the laws and to prosecutors. Some laws target either 'reckless' or 'negligent' transmission of or exposure to HIV. Others advocate criminalising only 'reckless' transmission of or exposure to HIV. We know that the 'reasonable person' often has unprotected sex with partners of unknown sexual history in spite of the known risks. That's why we have an HIV epidemic, and that's why interventions to reduce unsafe sex are so important.

When it comes to sex, with its potent elements of need, want, trust, passion, shame, fear, risk and heedlessness, normal, reasonable people simply do not always follow public health guidelines. With the best of intentions, they may make assumptions (e.g. suggesting condom use = "I am HIV+"), avoid issues (e.g. "no need to disclose if we just do oral sex"), or just hope for the best. HIV is a risk, but it is balanced in both parties' minds by the possibility of pleasure, excitement, closeness, material or social gain, and maybe love. That, for better or worse, is customary – yes, reasonable – behaviour.

But in court, looking back (especially looking back at an encounter where the worst outcome happened), a different standard is applied. As Matthew Weait's insightful account of British prosecutions has shown [14], the risk of HIV is treated as inherently unreasonable, and the decision of the putative victim to run the risk is rendered irrelevant by doctrines that require disclosure.

It is simply unfair to judge people, particularly a more or less arbitrarily selected small segment of the population, by legal standards of sexual behaviour that bear little relation to the standards of behaviour in real life.

Seventh, many of these laws are extremely poorly drafted. This is partly because of evidentiary burdens and the difficulty of satisfying them (that is, who infected whom). Because it is difficult to prove an offence that involves consensual sex, and because of the difficulties of applying the categories of the criminal law, many of these laws end up being a hodge-podge of confused legislative intent and bad drafting.

For instance, under the 'model law' that many countries in East and West Africa have adopted, a person who is aware of being infected with HIV must inform 'any sexual contact in advance' of this fact [15]. But the law does not say what 'any sexual contact' is. Is it holding hands? Kissing? Or only more intimate forms of exploratory contact? Or does it apply only to penetrative intercourse? The legal provision remains mysterious on these crucial issues.

What it also does not say is what 'in advance' means. Must it be before any sexual contact is initiated? Or is it only before actual intercourse occurs? Will people be prosecuted for intimate conduct intended to lead up to intercourse? We do not know. The laws do not say. Worse, millions of West and East Africans who must now live their lives under fear of prosecution by this law do not know.

The 'model' law would not pass muster in any constitutional state where the rule of law applies. The rule of law requires clarity in advance on the meaning of criminal provisions and the boundaries of criminal liability. But who will venture to challenge the laws as they have been enacted in 11 countries (as well as the often problematic laws criminalising HIV transmission and exposure in North America and Western Europe)? Until challenged, the terrifyingly vague provisions remain on the statute books.

Eighth, and perhaps most painfully to those living with HIV, criminalisation increases stigma. From the first diagnosis of AIDS 27 years ago, HIV has carried a mountainous burden of stigma. This has been for one over-riding reason: the fact that it is sexually transmitted. No other infectious disease is viewed with as much fear and repugnance as HIV. Because of this, stigma lies at the heart of the experience of every person living with or at risk of HIV.

It is stigma that makes those at risk of HIV reluctant to be tested; it is stigma that makes it difficult – and often impossible – for them to speak about their infection; and it is stigma that continues to hinder access to the life-saving antiretroviral therapies that are now increasingly available across Africa.

Legislators bewildered, baffled, or at a loss as to how to respond effectively to the epidemic may be seduced into taking recourse to criminalisation, because it seems attractive, effective and media-friendly. But it is not prevention- or treatment-friendly. It is hostile to both.

This is because, tragically, it is stigma that lies primarily behind the drive to criminalisation. It is stigma, rooted in the moralism that arises from the sexual transmission of HIV, which too often provides the main impulse behind the enactment of these laws.

Even more tragically, such laws and prosecutions in turn only add fuel to the fires of stigma. Prosecutions for HIV transmission and exposure, and the chilling content of the enactments themselves, reinforce the idea of HIV as a shameful, disgraceful, unworthy condition, requiring isolation and ostracism.

But HIV is a virus, not a crime. That fact is elementary, and all-important. Law-makers and prosecutors overlook it. We must fight this new burden of moralising stigma and persuade them of how wrong their approach is.

Ninth, criminalisation is a blatant disinducement to testing. It is radically incompatible with a public health strategy that seeks to encourage people to come forward to find out their HIV status. AIDS is now a medically manageable disease. Across Africa, the life-saving drugs that suppress the virus and restore the body to health are becoming increasingly available. But why should any woman in Kenya want to find out her HIV status, when her knowledge can only expose her to risk of prosecution? The laws put diagnosis, treatment, help and support further out of her reach.

By reinforcing stigma, by using the weapons of fear and blame and recrimination, criminalisation makes it more difficult for those with or at risk of HIV to access testing, to talk about diagnosis with HIV, and to receive treatment and support. We therefore have a dire but unavoidable calculus: these laws will lead to more deaths, more suffering and greater debilitation from AIDS. This when we need, instead, interventions that support openness and disclosure, and that help protect those with HIV from the stigma, discrimination and violence that may result. Criminal legislation cannot and will not assist.

Criminalisation is thus costing lives. The International Community of Women Living with HIV/AIDS has rightly described laws like this as part of a 'war on women' [16]. However, they are not just a war on women. They are a war on all people living with HIV, and they constitute an assault on good sense and rationality in dealing with the epidemic. The rush to legislation has resulted in rash, inappropriate and in all too many cases excessive laws. The laws often constitute an assault not just on civil liberties, but on rational and effective interventions in the epidemic.

And this brings us to the tenth and last point, which is about belief, and hope – words all too seldom heard in this epidemic. Criminalisation assumes the worst about people with HIV, and in doing so it punishes vulnerability. The human rights approach assumes the best about people with HIV and supports empowerment (see additional file 2) [17,18].

As Australian Justice Michael Kirby – who powerfully lights a pathway of justice and hope and reason in this epidemic – has argued, countries with human rights laws that encourage the undiagnosed to test for HIV do much better at containing the epidemic than those that have 'adopted punitive, moralistic, denialist strategies, including those relying on the criminal law as a sanction' [19].

The prevention of HIV is not just a technical challenge for public health. It is a challenge to all humanity to create a world in which behaving safely is truly feasible, is safe for both sexual partners, and genuinely rewarding. When condoms are available, when women have the power to use them, when those with HIV or at risk of it can get testing and treatment, when they are not afraid of stigma, ostracism and discrimination, they are far more likely to be able to act consistently for their own safety and that of others.

The global consensus on human rights and the need for an enabling environment captures this positive vision of HIV prevention. When compared with the punitive and angry approach embodied in criminalisation, the human rights-based approach is clearly more important now than ever. The principal effect of criminalisation is to enhance stigma, fear, isolation, and the dread of persecution and ostracism that drives people away from treatment.

In conclusion, we submit that:

• criminalisation is a poor tool for addressing HIV infection and transmission;

• there is no public health rationale for invoking criminal law sanctions against those who unknowingly and unintentionally transmit HIV or expose others to it;

• the sole rationale for criminalisation is the criminal law goal of retribution and punishment – but that is a poor and distorted aim for public health purposes; and

• criminalisation is in general warranted only in cases where someone sets out, well knowing he has HIV, to infect another person, and achieves this aim.

In other cases, we are left with the sad burdens, but also the hopeful initiatives, that are available to us in this epidemic. These include a resolve to fight stigma and discrimination, to counter criminalisation, and to fight instead for justice, good sense, effective prevention measures, gender equality, and for access to effective prevention and to treatment.

## Competing interests

The authors declare that they have no competing interests.

## Authors' contributions

The authors participated equally in the development of the arguments in the essay. EC wrote the first draft which was edited by SB and MC. All authors read and approved the final manuscript.

## Supplementary Material

Additional file 1 **Comment 1.** Additional comment for [12] Click here for file

Additional file 2 **Comment 2.** Additional comment for [18] Click here for file
