# Supplementary material for: HIV is a virus, not a crime: ten reasons against criminal statutes and criminal prosecutions
Source: J Int AIDS Soc. 2008 Dec 1;11:7. doi: 10.1186/1758-2652-11-7 (PMC2635346; doi:10.1186/1758-2652-11-7)
Supplement: Additional file 1 — Comment 1. Additional comment for [12] [file 1758-2652-11-7-S1.doc]

Comment 1

In West, Central and East Africa, Benin, Burundi, Burkina Faso, Cabo Verde, Central African Republic, Chad, the Democratic Republic of Congo, Equatorial Guinea, Guinea, Guinea Bissau, Kenya, Mali, Mauritania, Niger, Sierra Leone and Togo have adopted similar laws, as has Tanzania. We are grateful to Richard Pearshouse of the Canadian HIV/AIDS Legal Network for updating this information to 31 July 2008. Criminal prohibitions targeting HIV already exist, according to the AIDS and Rights Alliance of Southern Africa (ARASA), in Lesotho, Swaziland and Zimbabwe, with calls to criminalise transmission of HIV in Namibia and Zambia (and Uganda). In Lesotho, the Sexual Offences Act of 2003 criminalises wilful HIV transmission, as well as consensual sex where one of the parties fails to disclose his or her HIV status.
