# Supplementary material for: HIV is a virus, not a crime: ten reasons against criminal statutes and criminal prosecutions
Source: J Int AIDS Soc. 2008 Dec 1;11:7. doi: 10.1186/1758-2652-11-7 (PMC2635346; doi:10.1186/1758-2652-11-7)
Supplement: Additional file 2 — Comment 2. Additional comment for [18] [file 1758-2652-11-7-S2.doc]

Comment 2

In an address to an international criminal law reform conference in Dublin in July 2008, Justice Kirby argued that ‘Those countries that have adopted a human rights-respecting approach to the HIV/AIDS epidemic have been far more successful in containing the spread of HIV than those countries that have adopted punitive, moralistic, denialist strategies, including those relying on the criminal law as a sanction’.
